# Efficacy and safety of teneligliptin in patients with type 2 diabetes mellitus: a Bayesian network meta-analysis

**DOI:** 10.3389/fendo.2023.1282584

**Published:** 2023-12-18

**Authors:** Miao Zhu, Ruifang Guan, Guo Ma

**Affiliations:** Department of Clinical Pharmacy, School of Pharmacy, Fudan University, Shanghai, China

**Keywords:** teneligliptin, type 2 diabetes mellitus, systematic review, bayesian network meta-analysis, efficacy, safety

## Abstract

**Background:**

As a popular antidiabetic drug, teneligliptin has been used for over 10 years, but its efficacy and safety have rarely been systematically evaluated. Therefore, a Bayesian network meta-analysis was conducted to evaluate the efficacy and safety of teneligliptin in patients with type 2 diabetes mellitus (T2DM).

**Methods:**

We systematically searched PubMed, Web of Science, Embase, Cochrane Central Register of Controlled Trials, and ClinicalTrials.gov. Randomized controlled trials (RCTs) comparing teneligliptin with placebo or active comparators in T2DM patients for at least 12 weeks were included in the study. Data analysis was performed using R 4.2.3 and Stata 17.0 software. Each outcome was presented as a mean difference (MD) or an odds ratio (OR) along with 95% confidence interval (CI) and the surface under the cumulative ranking curve value (SUCRA).

**Results:**

A total of 18 RCTs with 3,290 participants with T2DM were included in this study. Generally, compared to placebo, sitagliptin, vildagliptin, metformin, and bromocriptine, 20 mg of teneligliptin showed better efficacy in reducing HbA1c (MD [95% CI], −0.78 [−0.86 to −0.70], −0.08 [−0.36 to 0.19], −0.04 [−0.72 to 0.60], −0.12 [−0.65 to 0.42], and −0.50 [−0.74 to −0.26], respectively) and fasting plasma glucose (FPG) (MD [95% CI], −18.02 [−20.64 to −15.13], 1.17 [−9.39 to 11.70], −8.06 [−30.95 to 14.35], −2.75 [−18.89 to 13.01], and −34.23 [−45.93 to −22.96], respectively), and 40 mg of teneligliptin also showed better efficacy in reducing HbA1c (MD [95% CI], −0.84 [−1.03 to −0.65], −0.15 [−0.49 to 0.19], −0.10 [−0.81 to 0.57], −0.18 [−0.76 to 0.39], and −0.56 [−0.88 to −0.26], respectively) and FPG (MD [95% CI], −20.40 [−26.07 to −14.57], −1.20 [−13.21 to 10.38], −10.43 [−34.16 to 12.65], −5.13 [−22.21 to 11.66], and −36.61 [−49.33 to −24.01], respectively). Compared to placebo, 20 mg of teneligliptin showed no significant difference in incidences of hypoglycemia and gastrointestinal adverse events (OR [95% CI], 1.30 [0.70 to 2.19] and 1.48 [0.78 to 2.98], respectively), and 40 mg of teneligliptin showed no significant difference in incidence of hypoglycemia (OR [95% CI], 2.63 [0.46 to 8.10]). Generally, antidiabetic effect and hypoglycemia risk of teneligliptin gradually increased as its dose increased from 5 mg to 40 mg. Compared to 20 mg of teneligliptin, 40 mg of teneligliptin showed superior efficacy and no-inferior safety, which was considered as the best option in reducing HbA1c, FPG, and 2h PPG and increasing proportion of the patients achieving HbA1c < 7% (SUCRA, 85.51%, 84.24%, 79.06%, and 85.81%, respectively) among all the included interventions.

**Conclusion:**

Compared to sitagliptin, vildagliptin, metformin, bromocriptine, and placebo, teneligliptin displayed favorable efficacy and acceptable safety in treating T2DM. Twenty milligrams or 40 mg per day was the optimal dosage regimen of teneligliptin. The results of this study will provide important evidence-based basis for rational use of teneligliptin and clinical decision-making of T2DM medication.

## 1 Introduction

Diabetes mellitus (DM) is a chronic metabolic disease mainly characterized by hyperglycemia, which seriously endangers human life and health. It is estimated that 537 million adults suffer from DM in the world at present, and this number is projected to increase to 783 million by 2045 (1, 2). Prevalence of DM is high, but its treatment rate and cure rate are low (3). Type 2 diabetes mellitus (T2DM) accounts for nearly 90% of DM in the world (4). The primary pathophysiology of T2DM is characterized by defective insulin secretion and insulin resistance (5). T2DM is mainly caused by a combination of genetic, metabolic, and environmental factors (6, 7). It is associated with increased risk of cardiovascular and renal outcomes under the long-term suboptimal glycemic control (8–10).

In recent years, some new antidiabetic drugs, such as dipeptidyl peptidase-4 inhibitors (DPP-4is), sodium-glucose cotransporter-2 inhibitors (SGLT-2is), and glucagon-like peptide-1 receptor agonists (GLP-1RAs), have been widely used in the treatment of T2DM (11). Of them, DPP-4is universally increase insulin secretion, and decrease levels of intact glucagon in patients with diabetes via potentiation of GLP-1 action (12). DPP-4is are widely welcomed by T2DM patients because of their excellent efficacy and safety.

As an oral DPP-4i launched in recent years, teneligliptin was approved as a treatment option for T2DM patients who have failed to control the blood glucose level under diet and exercise treatment in Japan (2012), Korea (2016), Thailand (2020), and China (2021) (13). It can significantly decrease the glycated hemoglobin A1c (HbA1c) and fasting plasma glucose (FPG) levels, and had a slight influence on body weight (BW). Twenty milligrams once daily is the current recommended dosage regimen for teneligliptin. In Japan, it is also being practiced with close monitoring to increase the dose of teneligliptin to 40 mg per day (14). Therefore, it needs to be further evaluated whether 40 mg of teneligliptin for T2DM patients is effective and safe or not.

It is crucial for rational application of antidiabetic drugs (e.g., teneligliptin) and precise drug treatment of DM by evaluating their efficacy and safety using some scientific methods. Network meta-analysis is a popular method to evaluate multiple treatments or interventions, and has usually been performed by the Bayesian approach (15, 16). Bayesian network meta-analysis is an effective method to simultaneously compare multiple treatments by combining the direct and indirect evidence, and it can provide results of relative rankings of different interventions (17, 18). The aim of this study was to evaluate the efficacy and safety of the DPP-4i teneligliptin in patients with T2DM by Bayesian network meta-analysis. The results of this study will provide important evidence-based basis for rational use of teneligliptin and clinical decision-making of T2DM medication.

## 2 Materials and methods

The Bayesian network meta-analysis was in accordance with the Preferred Reporting Items for Systematic Reviews and Meta-Analyses (PRISMA) guidelines and its extension for Network Meta-Analysis (19, 20).

### 2.1 Search strategy

The literature search was conducted in PubMed, Web of Science, Embase, Cochrane Central Register of Controlled Trials, and ClinicalTrials.gov from their inception to 22 March 2023, without language restriction. The databases were searched with the following MeSH (Medical Subject Headings) terms or keywords: (1) “Teneligliptin” OR “MP-513”; AND (2) “diabetes mellitus, type 2” OR “diabetes mellitus, type II” OR “noninsulin dependent diabetes” OR “non-insulin dependent diabetes” OR “NIDDM” OR “type II diabetes” OR “type 2 diabetes” OR “T2DM” OR “mature onset diabetes” OR “late onset diabetes” OR “adult onset diabetes”. Furthermore, the reference lists of identified trials were screened to further identify eligible trials.

### 2.2 Study selection

The concrete inclusion criteria were as follows: (1) Patients: any ethnicity, either gender, aged 18 years or older, and HbA1c ≥ 6.5%. (2) Interventions: any dose of teneligliptin used as monotherapy or combination therapy with duration of at least 12 weeks. (3) Comparison: placebo or active comparators with or without background therapy. (4) Outcomes: at least one of the following indicators was reported: HbA1c, the patients achieving HbA1c < 7%, FPG, 2 h postprandial plasma glucose (2h PPG), BW, body mass index (BMI), hypoglycemia, and gastrointestinal adverse events (GIAEs). (5) Study design: randomized controlled trials (RCTs) published without language restrictions.

The studies were excluded if they included patients of age over 75 years, with HbA1c>10%, and with a history of renal, hepatic failures, dyslipidemia, or cardiovascular disorders. Moreover, phase I studies and secondary analyses were excluded.

### 2.3 Data extraction

The following information from the included studies were extracted: study information, baseline characteristics of the patient, intervention measures, and prespecified outcomes. For all the outcomes, we extracted data for the intention-to-treat (ITT) population, which comprised all the randomly assigned patients who received at least one dose of the study medication.

### 2.4 Risk-of-bias assessment

Assessment of the risk of bias was conducted by Cochrane Review Manager (RevMan), which included selection, performance, detection, attrition, and reporting bias (21). The scores for each aspect of eligible studies were recorded as high, low, or unclear risk.

The study search and selection, data extraction, and risk of bias assessment were conducted independently by two reviewers (MZ and RFG). Any differences were resolved through discussion or consultation with a third independent reviewer (GM).

### 2.5 Statistical analysis

A network meta-analysis with Bayesian approach was performed using R version 4.2.3 (http://www.r-project.org/) with the packages GEMTC, RJAGS, EXPORT, and BUGSnet and Stata MP 17.0 (StataCrop LLC) in this study (22, 23).

#### 2.5.1 Synthesis of treatments and outcomes

For each prespecified outcome, a network plot of all the interventions was made to identify possible direct and indirect comparisons. The width of the lines in the network plot is proportional to the number of studies, and the node sizes correspond to number of the participants achieving a certain treatment in the comparisons. Effect estimates included odds ratio (OR) for categorical outcomes and mean difference (MD) for continuous outcomes. If the standard deviation (SD) was not reported, it was calculated from the standard error (SE), probability (*p*) value, confidence interval (CI), or MD according to the guidance from the Cochrane Handbook for Systematic Reviews of Interventions (24).

#### 2.5.2 Model fitting and consistency evaluation

The Markov chain Monte Carlo algorithm was used for each outcome based on 20,000 simulation iterations and 5,000 adaptation iterations. A thinning interval of 1 was applied, which collected one sample every one iteration. The selection between fixed and random model and the evaluation of model fit goodness were realized through deviance information criteria (DIC), and the model with a lower value was chosen. The difference value of DIC between inconsistency and consistency models under 3 indicates a good consistency of network meta-analysis.

#### 2.5.3 Effectiveness evaluation

The difference between the comparisons was considered statistically significant when the 95% CI did not contain 0.00 for continuous outcomes or 1.00 for categorical outcomes. The point estimates with 95% CI for each treatment comparison were presented in a league table. The surface under the cumulative ranking curve (SUCRA), showing each intervention ranking with respective ranking possibility, was calculated to rank the efficacy of each treatment. The SUCRA values ranged from 0% to 100%. The higher value indicates that a particular treatment is more possible to be in a top rank; similarly, the lower value indicates that a particular treatment is more possible to be in a bottom rank (25).

#### 2.5.4 Heterogeneity evaluation and publication bias

*I* ^2^ statistic was used to assess the heterogeneity arising from differences between the studies within each direct comparison of treatments, and the value of >50% indicated significant heterogeneity between the studies. Publication bias was evaluated using funnel plots.

## 3 Results

### 3.1 Study selection and characteristics

**Figure 1:**
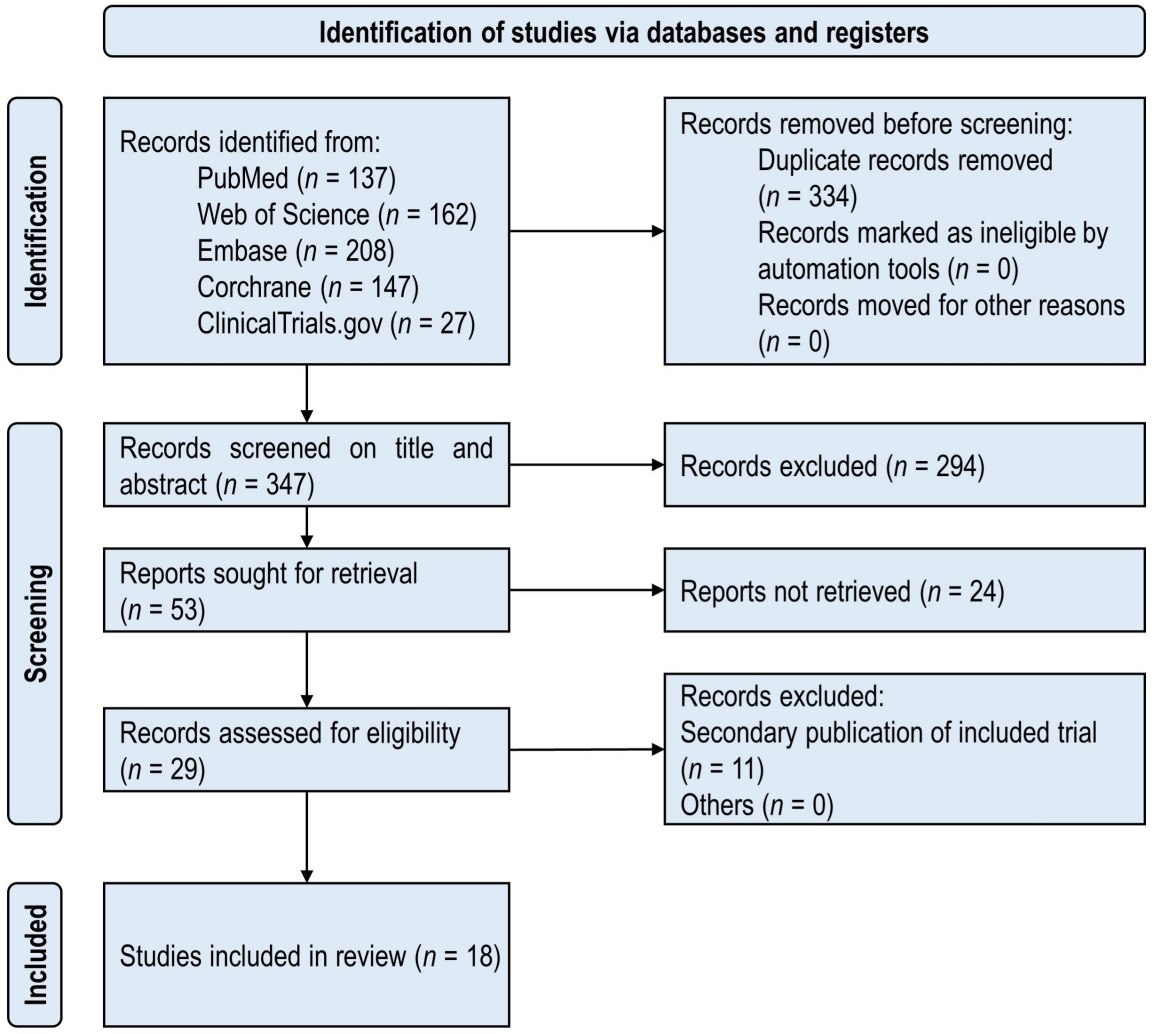
PRISMA flowchart of the included studies.

The PRISMA flowchart of the included studies is shown in **Figure 1**. There were 681 publications initially identified in five databases. A total of 18 unique RCTs (26–43) met the inclusion criteria and were considered for the proposed study. The included studies were published from 2013 to 2023, which consisted of 3,290 participants with T2DM in total. The minimum number of participants was 40, and the maximum number was 447 in the 18 included RCTs.

The detailed characteristics of the included studies are provided in **Table S1**. The weighted means of age, BW, BMI, baseline HbA1c, and baseline FPG were 56.6 years, 73.5 kg, 26.7 kg/m^2^, 8.0%, and 156.0 mg/dL, respectively. Compared with the others, only one study (33) stood out in terms of BW and BMI (weighted means were 92.8 kg and 32.3 kg/m^2^, respectively). These differences did not have a significant impact on similarity of baseline characteristics of the included studies. The duration of treatment ranged from 12 to 24 weeks.

**Figure 2:**
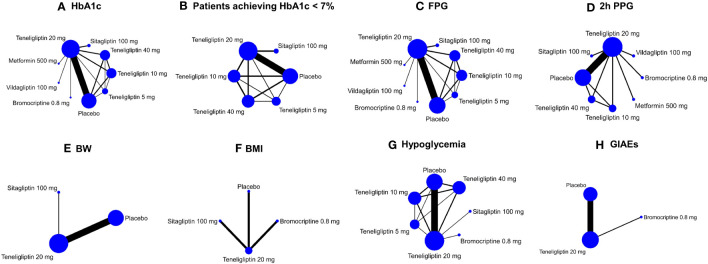
Network plots of efficacy and safety outcomes. These network plots show comparisons of teneligliptin, sitagliptin, vildagliptin, metformin, bromocriptine, and placebo in the RCTs with respect to the sample sizes and number of studies according to eight different outcome measures. The doses of all the antidiabetic drugs are daily dose. Each node represents a certain intervention, and its size represents number of the participants given a certain intervention in the comparisons. The width of the lines represents the number of studies comparing every pair of interventions. HbA1c, glycated hemoglobin A1c; FPG, fasting plasma glucose; 2h PPG, 2 h postprandial plasma glucose; BW, body weight; BMI, body mass index; GIAEs, gastrointestinal adverse events. **(A)** HbA1c; **(B)** Patients achieving HbA1c < 7%; **(C)** FPG; **(D)** 2h PPG; **(E)** BW; **(F)** BMI; **(G)** Hypoglycemia; **(H)** GIAEs.

Included in the meta-analysis were the following doses: 5 mg, 10 mg, 20 mg, and 40 mg of teneligliptin (qd), 100 mg of sitagliptin (qd), 50 mg of vildagliptin (bid), 500 mg of metformin (qd), and 0.8 mg of bromocriptine (qd). Efficacy outcomes contained mean changes of HbA1c, FPG, 2h PPG, BW, and BMI as well as proportion of the patients achieving HbA1c < 7%. Safety outcomes included incidences of hypoglycemia and GIAEs. Outcomes of HbA1c and FPG were reported in all the 18 included studies. The other six outcomes were only reported in a part of the 18 included studies (**Table S1**). Network plots of all the efficacy and safety outcomes are illustrated in **Figure 2**.

### 3.2 Risk-of-bias analysis

**Figure 3:**
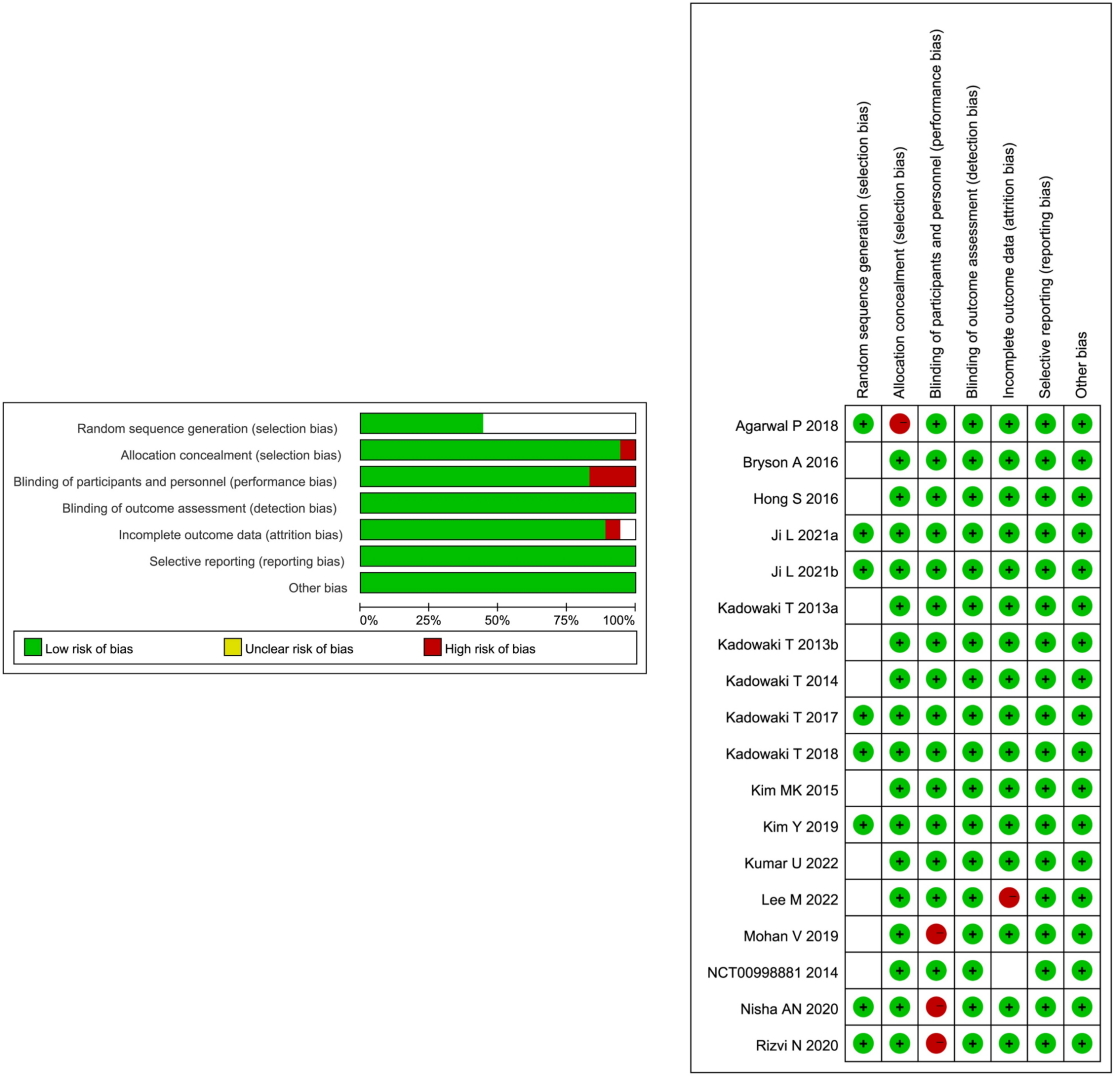
Assessment of the risk of bias in the included studies.

The risk of bias in the 18 included RCTs is summarized in **Figure 3**. Among the 18 RCTs, all of them had low risk for bias of blinding of outcome assessment, incomplete outcome data, and selective reporting, 8 RCTs had low risk for bias in random sequence generation, and the other 10 RCTs had unclear risk of bias, 17 RCTs had low risk for bias in allocation concealment, 15 RCTs had low risk for bias in blinding of participants and personnel, 16 RCTs had low risk for bias in incomplete outcome data, and 1 RCT had unclear risk. Overall, these studies had a low or moderate level of risk.

### 3.3 Network meta-analysis

**Figure 4:**
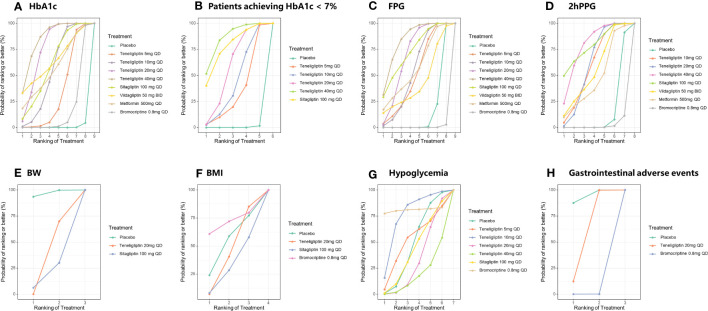
SUCRA plots of efficacy and safety outcomes. SUCRA, the surface under the cumulative ranking curve value; HbA1c, glycated hemoglobin A1c; FPG, fasting plasma glucose; 2h PPG, 2 h postprandial plasma glucose; BW, body weight; BMI, body mass index; GIAEs, gastrointestinal adverse events. **(A)** HbA1c; **(B)** Patients achieving HbA1c < 7%; **(C)** FPG; **(D)** 2h PPG; **(E)** BW; **(F)** BMI; **(G)** Hypoglycemia; **(H)** GIAEs.

The statistical analysis of all indicators was performed using a random-effects model. SUCRA values of all the interventions and outcomes are shown in **Figure 4** and **Table 1**. League tables of the efficacy outcomes (HbA1c, proportion of the patients achieving HbA1c < 7%, FPG, 2h PPG, BW, and BMI) are shown in **Tables 2**–**4**. League table of the safety outcomes (hypoglycemia, GIAEs) is shown in **Table 5**.

**Table 1:** SUCRA (%) of various interventions.

Interventions	HbA1c		Patients achieving HbA1c < 7%		FPG		2h PPG		BW		BMI		Hypoglycemia		GIAEs	
	SUCRA	Rank	SUCRA	Rank	SUCRA	Rank	SUCRA	Rank	SUCRA	Rank	SUCRA	Rank	SUCRA	Rank	SUCRA	Rank
Placebo	0.57	9	0.33	6	15.37	8	14.20	7	96.67	1	53.35	2	48.22	4	93.75	1
Bromocriptine 0.8 mg	16.18	8	NA	NA	0.38	9	1.93	8	NA	NA	70.87	1	80.82	1	0.15	3
Metformin 500 mg	57.48	5	NA	NA	57.45	4	48.07	6	NA	NA	NA	NA	NA	NA	NA	NA
Vildagliptin 100 mg	64.41	3	NA	NA	43.29	7	56.11	5	NA	NA	NA	NA	NA	NA	NA	NA
Sitagliptin 100 mg	61.30	4	77.85	2	73.50	2	79.06	1	18.25	3	31.48	4	42.70	5	NA	NA
Teneligliptin 5 mg	33.66	7	34.33	5	51.45	6	NA	NA	NA	NA	NA	NA	51.44	3	NA	NA
Teneligliptin 10 mg	55.21	6	43.60	4	54.80	5	60.25	4	NA	NA	NA	NA	75.66	2	NA	NA
Teneligliptin 20 mg	75.67	2	58.07	3	69.53	3	61.28	3	35.07	2	44.31	3	32.82	6	56.11	2
Teneligliptin 40 mg	85.51	1	85.81	1	84.24	1	79.06	2	NA	NA	NA	NA	18.35	7	NA	NA

The doses of all the antidiabetic drugs are daily dose. SUCRA, the surface under the cumulative ranking curve value; FPG, fasting plasma glucose; 2h PPG, 2 h postprandial plasma glucose; BW, body weight; BMI, body mass index; GIAEs, gastrointestinal adverse events; NA, not available.

**Table 2:** League table of HbA1c (upper right quarter) and proportion of the patients achieving HbA1c < 7% (lower left quarter).

**Placebo**	−0.49 (−0.73, −0.26)	−0.66 (−0.86, −0.48)	−0.78 (−0.86, −0.70)	−0.84 (−1.03, −0.65)	−0.69 (−0.98, −0.40)	−0.74 (−1.38, −0.05)	−0.66 (−1.20, −0.12)	−0.27 (−0.53, −0.02)
0.30 (0.07, 0.83)	**Teneligliptin** **5 mg**	−0.17 (−0.42, 0.08)	−0.28 (−0.51, −0.05)	−0.35 (−0.60, −0.10)	−0.20 (−0.56, 0.17)	−0.24 (−0.93, 0.48)	−0.17 (−0.75, 0.45)	0.22 (−0.11, 0.55)
0.22 (0.08, 0.49)	0.99 (0.21, 2.92)	**Teneligliptin** **10 mg**	−0.11 (−0.30, 0.08)	−0.18 (−0.39, 0.04)	−0.03 (−0.36, 0.32)	−0.07 (−0.76, 0.63)	0.00 (−0.57, 0.60)	0.39 (0.09, 0.70)
0.16 (0.09, 0.24)	0.74 (0.18, 2.07)	0.87 (0.30, 1.93)	**Teneligliptin** **20 mg**	−0.06 (−0.25, 0.12)	0.08 (−0.19, 0.36)	0.04 (−0.60, 0.72)	0.12 (−0.42, 0.65)	0.50 (0.26, 0.74)
0.11 (0.04, 0.24)	0.48 (0.10, 1.39)	0.56 (0.17, 1.35)	0.70 (0.24, 1.58)	**Teneligliptin** **40 mg**	0.15 (−0.19, 0.49)	0.10 (−0.57, 0.81)	0.18 (−0.39, 0.76)	0.56 (0.26, 0.88)
0.13 (0.03, 0.33)	0.61 (0.08, 2.15)	0.71 (0.13, 2.20)	0.81 (0.24, 2.01)	1.47 (0.27, 4.68)	**Sitagliptin** **100 mg**	−0.04 (−0.78, 0.68)	0.03 (−0.58, 0.63)	0.42 (0.05, 0.78)
NA	NA	NA	NA	NA	NA	**Vildagliptin** **100 mg**	0.08 (−0.80, 1.00)	0.46 (−0.26, 1.14)
NA	NA	NA	NA	NA	NA	NA	**Metformin** **500 mg**	0.38 (−0.20, 0.98)
NA	NA	NA	NA	NA	NA	NA	NA	**Bromocriptine** **0.8 mg**

The relative effect sizes are measured as a mean difference (upper right quarter) or an odds ratio (lower left quarter) along with 95% CIs. The differences between the compared groups are deemed as significant when the 95% CIs did not contain 0.00 (upper right quarter) or 1.00 (lower left quarter). The results with significant differences are marked with blue background, and the results without significant differences are marked with gray background. The doses of all the antidiabetic drugs are daily dose. The unit of HbA1c is %. HbA1c, glycated hemoglobin A1c; NA, not available.

**Table 3:** League table of FPG (upper right quarter) and 2h PPG (lower left quarter).

**Placebo**	−14.87 (−23.34, −6.30)	−15.87 (−21.42, −9.88)	−18.02 (−20.64, −15.13)	−20.40 (−26.07, −14.57)	−19.20 (−29.90, −8.12)	−9.97 (−32.71, 13.08)	−15.27 (−31.23, 1.03)	16.21 (4.77, 28.36)
NA	**Teneligliptin** **5 mg**	−0.99 (−9.87, 8.05)	−3.15 (−11.65, 5.37)	−5.53 (−14.81, 3.49)	−4.32 (−17.68, 9.34)	4.90 (−18.41, 29.21)	−0.40 (−18.00, 17.98)	31.08 (17.22, 45.38)
45.09 (24.57, 64.93)	NA	**Teneligliptin** **10 mg**	−2.16 (−7.94, 3.47)	−4.54 (−11.12, 1.87)	−3.33 (−15.23, 8.54)	5.90 (−17.06, 29.54)	0.60 (−16.16, 17.68)	32.07 (19.50, 44.84)
45.61 (36.29, 53.97)	NA	0.52 (−19.81, 20.53)	**Teneligliptin** **20 mg**	−2.38 (−8.15, 3.41)	−1.17 (−11.70, 9.39)	8.06 (−14.35, 30.95)	2.75 (−13.01, 18.89)	34.23 (22.96, 45.93)
53.00 (32.80, 73.09)	NA	7.91 (−14.68, 30.84)	7.39 (−12.56, 27.84)	**Teneligliptin** **40 mg**	1.20 (−10.38, 13.21)	10.43 (−12.65, 34.16)	5.13 (−11.66, 22.21)	36.61 (24.01, 49.33)
58.79 (15.27, 104.65)	NA	13.70 (−33.42, 62.96)	13.18 (−29.91, 58.23)	5.79 (−41.47, 55.43)	**Sitagliptin** **100 mg**	9.23 (−16.00, 33.26)	3.93 (−15.30, 23.23)	35.40 (20.15, 51.02)
41.41 (5.33, 76.86)	NA	−3.68 (−43.67, 35.64)	−4.20 (−38.77, 30.03)	−11.59 (−52.29, 27.73)	−17.38 (−74.25, 38.24)	**Vildagliptin** **100 mg**	−5.30 (−32.85, 22.49)	26.17 (0.15, 51.36)
34.04 (−12.59, 77.95)	NA	−11.05 (−60.74, 36.33)	−11.57 (−56.89, 31.81)	−18.95 (−68.78, 28.22)	−24.75 (−88.81, 35.62)	−7.37 (−63.51, 48.31)	**Metformin** **500 mg**	31.48 (12.10, 50.80)
−15.31 (−41.06, 9.97)	NA	−60.40 (−91.62, −28.94)	−60.92 (−84.64, −36.67)	−68.31 (−99.66, −36.79)	−74.10 (−124.95, −24.55)	−56.72 (−98.61, −14.95)	−49.35 (−97.98, 1.49)	**Bromocriptine** **0.8 mg**

The relative effect sizes are measured as a mean difference along with 95% CIs. The differences between the compared groups are deemed as significant when the 95% CIs did not contain 0.00. The results with significant differences are marked with blue background, and the results without significant differences are marked with gray background. The doses of all the antidiabetic drugs are daily dose. The unit of FPG and 2h PPG is mg/dL. FPG, fasting plasma glucose; 2h PPG, 2 h postprandial plasma glucose; NA, not available.

**Table 4:** League table of BW (upper right quarter) and BMI (lower left quarter).

**Placebo**	0.58 (0.22, 0.92)	0.82 (-0.29, 1.94)	NA
−0.11 (−0.94, 0.72)	**Teneligliptin 20 mg**	0.25 (-0.82, 1.32)	NA
−0.20 (−1.25, 0.85)	−0.09 (−0.74, 0.57)	**Sitagliptin 100 mg**	NA
0.39 (−1.48, 2.23)	0.50 (−1.18, 2.16)	0.59 (−1.21, 2.35)	**Bromocriptine 0.8 mg**

The relative effect sizes are measured as a mean difference along with 95% CIs. The differences between the compared groups are deemed as significant when the 95% CIs did not contain 0.00. The results with significant differences are marked with blue background, and the results without significant differences are marked with gray background. The doses of all the antidiabetic drugs are daily dose. The unit of BW and BMI is kg and kg/m^2^, respectively. BW, body weight; BMI, body mass index; NA, not available.

**Table 5:** League table of hypoglycemia (upper right quarter) and GIAEs (lower left quarter).

**Placebo**	1.60 (0.03, 7.72)	0.51 (0.01, 2.27)	1.30 (0.70, 2.19)	2.63 (0.46, 8.10)	1.35 (0.30, 3.72)	3,867,030.67 (0.00, 400,117.91)
NA	**Teneligliptin** **5 mg**	2.13 (0.01, 16.00)	9.39 (0.16, 39.22)	21.77 (0.24, 75.01)	9.84 (0.10, 42.35)	5,377,343.72 (0.00, 947,506.86)
NA	NA	**Teneligliptin** **10 mg**	15.44 (0.53, 87.30)	26.63 (0.86, 149.16)	16.56 (0.34, 89.05)	7,695,257.57 (0.00, 1,838,505.19)
0.76 (0.34, 1.28)	NA	NA	**Teneligliptin** **20 mg**	2.13 (0.36, 6.88)	1.03 (0.28, 2.69)	2,438,052.60 (0.00, 340,812.34)
NA	NA	NA	NA	**Teneligliptin** **40 mg**	0.85 (0.09, 3.32)	2,299,670.23 (0.00, 224,503.51)
NA	NA	NA	NA	NA	**Sitagliptin** **100 mg**	4,025,460.15 (0.00, 433,075.76)
0.01 (0.00, 0.12)	NA	NA	0.02 (0.00, 0.16)	NA	NA	**Bromocriptine** **0.8 mg**

The relative effect sizes are measured as an odds ratio along with 95% CIs. The differences between the compared groups are deemed as significant when the 95% CIs did not contain 1.00. The results with significant differences are marked with blue background, and the results without significant differences are marked with gray background. The doses of all the antidiabetic drugs are daily dose. GIAEs, gastrointestinal adverse events; NA, not available.

#### 3.3.1 HbA1c

As shown in **Table 2**, compared to placebo, 5 mg, 10 mg, 20 mg, and 40 mg of teneligliptin showed significant efficacy in reducing HbA1c (MD [95% CI], −0.49 [−0.73 to −0.26], −0.66 [−0.86 to −0.48], −0.78 [−0.86 to −0.70], and −0.84 [−1.03 to −0.65], respectively). Compared to sitagliptin, vildagliptin, and metformin, 20 mg and 40 mg of teneligliptin showed better efficacy in reducing HbA1c (MD [95% CI], 20 mg: −0.08 [−0.36 to 0.19], −0.04 [−0.72 to 0.60], and −0.12 [−0.65 to 0.42]; 40 mg: −0.15 [−0.49 to 0.19], −0.10 [−0.81 to 0.57], and −0.18 [−0.76 to 0.39], respectively). Compared to sitagliptin, vildagliptin, and metformin, 5 mg and 10 mg of teneligliptin showed weaker efficacy in reducing HbA1c (MD [95% CI], 5 mg: 0.20 [−0.17 to 0.54], 0.24 [−0.48 to 0.93], and 0.17 [−0.45 to 0.75]; 10 mg: 0.03 [−0.32 to 0.36], 0.07 [−0.63 to 0.76], and 0.00 [−0.60 to 0.57], respectively). Compared to bromocriptine, 10 mg, 20 mg, and 40 mg of teneligliptin showed significant efficacy in reducing HbA1c (MD [95% CI], −0.39 [−0.70 to −0.09], −0.50 [−0.74 to −0.26], and −0.56 [−0.88 to −0.26], respectively).

The results of SUCRA indicated that 40 mg of teneligliptin was the best option in reducing HbA1c (85.51%), followed by 20 mg of teneligliptin (75.67%), 100 mg of vildagliptin (64.41%), 100 mg of sitagliptin (61.3%), 500 mg of metformin (57.48%), 10 mg of teneligliptin (55.21%), 5 mg of teneligliptin (33.66%), 0.8 mg of bromocriptine (16.18%), and placebo (0.57%) (**Figure 4A** and **Table 1**).

#### 3.3.2 Proportion of the patients achieving HbA1c < 7%

Compared to placebo, 5 mg, 10 mg, 20 mg, and 40 mg of teneligliptin showed significant efficacy in increasing the proportion of the patients achieving HbA1c < 7% (OR [95% CI], 4.95 [1.20 to 14.09], 5.74 [2.03 to 13.22], 6.81 [4.13 to 11.03], and 11.97 [4.19 to 28.48], respectively). Compared to sitagliptin, 40 mg of teneligliptin showed better efficacy in increasing the proportion of the patients achieving HbA1c < 7% (OR [95% CI], 1.47 [0.27 to 4.68]). Compared to sitagliptin, 5 mg, 10 mg, and 20 mg of teneligliptin showed weaker efficacy (OR [95% CI], 0.61 [0.08 to 2.15], 0.71 [0.13 to 2.20], and 0.81 [0.24 to 2.01], respectively) (**Table 2**).

The results of SUCRA indicated that, superiority of increasing proportion of the patients achieving HbA1c < 7% ranked as follows: 40 mg of teneligliptin (85.81%), 100 mg of sitagliptin (77.85%), 20 mg of teneligliptin (58.07%), 10 mg of teneligliptin (43.6%), 5 mg of teneligliptin (34.33%), and placebo (0.33%) (**Figure 4B** and **Table 1**).

#### 3.3.3 FPG

As shown in **Table 3**, compared to placebo, 5 mg, 10 mg, 20 mg, and 40 mg of teneligliptin showed significant efficacy in reducing FPG (MD [95% CI], −14.87 [−23.34 to −6.30], −15.87 [−21.42 to −9.88], −18.02 [−20.64 to −15.13], and −20.40 [−26.07 to −14.57], respectively). Compared to sitagliptin, 40 mg of teneligliptin showed better efficacy in reducing FPG (MD [95% CI], −1.20 [−13.21 to 10.38]). Compared to sitagliptin, 5 mg, 10 mg, and 20 mg of teneligliptin showed weaker efficacy (MD [95% CI], 4.32 [−9.34 to 17.68], 3.33 [−8.54 to 15.23], and 1.17 [−9.39 to 11.70], respectively). Compared to vildagliptin, 5 mg, 10 mg, 20 mg, and 40 mg of teneligliptin showed better efficacy in reducing FPG (MD [95% CI], −4.90 [−29.21 to 18.41], −5.90 [−29.54 to 17.06], −8.06 [−30.95 to 14.35], and −10.43 [−34.16 to 12.65], respectively). Compared to metformin, 10 mg, 20 mg, and 40 mg of teneligliptin showed better efficacy in reducing FPG (MD [95% CI], −0.60 [−17.68 to 16.16], −2.75 [−18.89 to 13.01], and −5.13 [−22.21 to 11.66], respectively). Compared to metformin, 5 mg of teneligliptin showed weaker efficacy (MD [95% CI], 0.40 [−17.98 to 18.00]). Moreover, compared to bromocriptine, 5 mg, 10 mg, 20 mg, and 40 mg of teneligliptin showed significant efficacy in reducing FPG (MD [95% CI], −31.08 [−45.38 to −17.22], −32.07 [−44.84 to −19.50], −34.23 [−45.93 to −22.96], and −36.61 [−49.33 to −24.01], respectively).

According to the SUCRA (**Figure 4C** and **Table 1**), 40 mg of teneligliptin (84.24%) seemed to be the most effective option in reducing FPG, followed by 100 mg of sitagliptin (73.5%), 20 mg of teneligliptin (69.53%), 500 mg of metformin (57.45%), 10 mg of teneligliptin (54.8%), 5 mg of teneligliptin (51.45%), 100 mg of vildagliptin (43.29%), placebo (15.37%), and 0.8 mg of bromocriptine (0.38%).

#### 3.3.4 2h PPG

Compared to placebo, 10 mg, 20 mg and 40 mg of teneligliptin showed significant efficacy in reducing 2h PPG (MD [95% CI], −45.09 [−64.93 to −24.57], −45.61 [−53.97 to −36.29], and −53.00 [−73.09 to −32.80], respectively). Compared to sitagliptin, 10 mg, 20 mg, and 40 mg of teneligliptin showed weaker efficacy in reducing 2h PPG (MD [95% CI], 13.70 [−33.42 to 62.96], 13.18 [−29.91 to 58.23], and 5.79 [−41.47 to 55.43], respectively). Compared to vildagliptin, 10 mg, 20 mg, and 40 mg of teneligliptin showed better efficacy in reducing 2h PPG (MD [95% CI], −3.68 [−43.67 to 35.64], −4.20 [−38.77 to 30.03], and −11.59 [−52.29 to 27.73], respectively). Compared to metformin, 10 mg, 20 mg, and 40 mg of teneligliptin also showed better efficacy in reducing 2h PPG (MD [95% CI], −11.05 [−60.74 to 36.33], −11.57 [−56.89 to 31.81], and −18.95 [−68.78 to 28.22], respectively). Compared to bromocriptine, 10 mg, 20 mg, and 40 mg of teneligliptin showed significant efficacy in reducing 2h PPG (MD [95% CI], −60.40 [−91.62 to −28.94], −60.92 [−84.64 to −36.67], and −68.31 [−99.66 to −36.79], respectively) (**Table 3**).

According to the SUCRA (**Figure 4D** and **Table 1**), both 100 mg of sitagliptin and 40 mg of teneligliptin seemed to be the best intervention in reducing 2h PPG (79.06%), followed by 20 mg of teneligliptin (61.28%), 10 mg of teneligliptin (60.25%), 100 mg of vildagliptin (56.11%), 500 mg of metformin (48.07%), placebo (14.2%), and 0.8 mg of bromocriptine (1.93%).

#### 3.3.5 BW

As shown in **Table 4**, 20 mg of teneligliptin showed better efficacy in reducing BW than sitagliptin (MD [95% CI], −0.25 [−1.32 to 0.82]). However, 20 mg of teneligliptin showed significantly weaker efficacy than placebo (MD [95% CI], 0.58 [0.22 to 0.92]). According to the SUCRA (**Figure 4E** and **Table 1**), 20 mg of teneligliptin (35.07%) showed a better effect in reducing BW than sitagliptin (18.25%).

#### 3.3.6 BMI

Compared to placebo, 20 mg of teneligliptin and 100 mg of sitagliptin showed weaker efficacy in reducing BMI (MD [95% CI], 0.11 [−0.72 to 0.94] and 0.20 [−0.85 to 1.25], respectively). Compared to placebo, 0.8 mg of bromocriptine showed better efficacy (MD [95% CI], −0.39 [−2.23 to 1.48]) (**Table 4**). According to the SUCRA, 0.8 mg of bromocriptine was the best option in reducing BMI (70.87%), followed by placebo (53.35%), 20 mg of teneligliptin (44.31%), and 100 mg of sitagliptin (31.48%) (**Figure 4F** and **Table 1**).

#### 3.3.7 Hypoglycemia

As shown in **Table 5**, compared to placebo, 5 mg, 10 mg, 20 mg, and 40 mg of teneligliptin showed no-inferior risk of hypoglycemia (OR [95% CI], 1.60 [0.03 to 7.72], 0.51 [0.01 to 2.27], 1.30 [0.70 to 2.19], and 2.63 [0.46 to 8.10], respectively). In addition, compared to placebo, sitagliptin and bromocriptine also showed no significant difference in incidence of hypoglycemia. According to the SUCRA (**Figure 4G** and **Table 1**), 0.8 mg of bromocriptine (80.82%) was considered as the best intervention in avoiding hypoglycemia, followed by 10 mg of teneligliptin (75.66%), 5 mg of teneligliptin (51.44%), placebo (48.22%), 100 mg of sitagliptin (42.70%), 20 mg of teneligliptin (32.82%), and 40 mg of teneligliptin (18.35%).

#### 3.3.8 GIAEs

Compared to placebo, 20 mg of teneligliptin showed no significant difference in incidence of GIAEs (OR [95% CI], 1.48 [0.78 to 2.98]). Compared to bromocriptine, 20 mg of teneligliptin had a significantly lower risk of GIAEs (OR [95% CI], 0.02 [0.00 to 0.16]) (**Table 5**). According to the SUCRA (**Figure 4H** and **Table 1**), 20 mg of teneligliptin (56.11%) had a lower incidence of GIAEs than 0.8 mg of bromocriptine (0.15%).

#### 3.3.9 Consistency and heterogeneity tests

Difference value of DIC between inconsistency and consistency models for each outcome was less than three, indicating that inconsistency of this network analysis was not significant. For most outcome measures of this study, *I* ^2^ statistic value was under 50%, and heterogeneity was not obvious. Only *I* ^2^ values of two outcomes (i.e., BW and BMI) were over 50%. According to the sensitivity analysis (**Table S2**), the *I* ^2^ value of network analysis of BW outcome decreased to 42.62% (<50%) after eliminating one (35) of the included studies. However, for network analysis of BMI outcome, the heterogeneity was obvious with a *I* ^2^ value > 50% after eliminating one of the included studies (36, 38, 43) in turn.

#### 3.3.10 Publication bias

**Figure 5:**
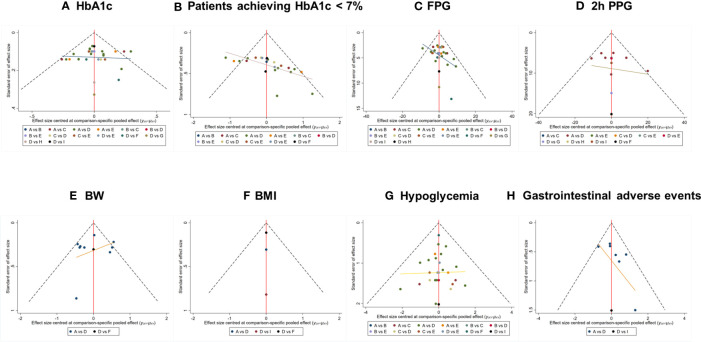
Comparison-adjusted funnel plot for outcomes. The funnel plots displayed publication bias of respective outcomes: **(A)** HbA1c, **(B)** Patients achieving HbA1c < 7%, **(C)** FPG, **(D)** 2h PPG, **(E)** BW, **(F)** BMI, **(G)** Hypoglycemia, **(H)** GIAEs. The different colored nodes in the plots represent certain paired comparisons of respective interventions: **(A)** Placebo, **(B)** 5 mg of teneligliptin, **(C)** 10 mg of teneligliptin, **(D)** 20 mg of teneligliptin, **(E)** 40 mg of teneligliptin, **(F)** 100 mg of sitagliptin, **(G)** 100 mg of vildagliptin, **(H)** 500mg of metformin, **(I)** 0.8 mg of bromocriptine. HbA1c, glycated hemoglobin A1c; FPG, fasting plasma glucose; 2h PPG, 2 h postprandial plasma glucose; BW, body weight; BMI, body mass index; GIAEs, gastrointestinal adverse events.

The comparison-adjusted funnel plots for assessment of publication bias are shown in **Figure 5**. Visual inspections indicated that, distribution of the included studies was not asymmetric, and there was some angle between the adjusted auxiliary line and the horizontal zero line, suggesting that some publication bias may exist.

## 4 Discussion

Compared to placebo, 5 mg, 10 mg, 20 mg, and 40 mg of teneligliptin were better in most efficacy outcomes except for reducing BW and BMI. This indicated that teneligliptin showed observable glucose-lowering and poor weight-loss effect. In all safety outcomes, there was no significant difference among placebo and the four doses of teneligliptin. This implied that teneligliptin showed acceptable safety. It should be noted that the heterogeneity in the network meta-analyses of BW and BMI were obvious, so their reliability was lower than the other outcome measures.

Efficacy of teneligliptin increased with its dose from 5 mg to 40 mg, but the risk of hypoglycemia also increased. In particular, 10 mg of teneligliptin showed the lowest risk of hypoglycemia among the four doses. Compared to 20 mg of teneligliptin, 40 mg of teneligliptin showed superior glucose-lowering efficacy and no-inferior safety. Therefore, it is a favorable option to increase the dose of teneligliptin from 20 mg to 40 mg per day when its antidiabetic effect is not satisfactory. It should be noted that the limitation of this study was that 40 mg of teneligliptin was only presented in two RCTs with 169 patients in this meta-analysis, and more clinical trials with more patients should be conducted to further confirm this result in the future.

Compared to the single dose of sitagliptin and vildagliptin, four doses of teneligliptin showed a different antidiabetic effect. Among these included DPP-4is, their antidiabetic effects ranked as follows: 40 mg of teneligliptin, 20 mg of teneligliptin, 100 mg of sitagliptin, 100 mg of vildagliptin, 10 mg of teneligliptin, and 5 mg of teneligliptin. It should be noted that a lower dose of teneligliptin showed better antidiabetic effect than sitagliptin and vildagliptin. Additionally, there was no significant difference among the four doses of teneligliptin and sitagliptin in all safety outcomes. The results can provide reference for the rational selection among these DPP-4is.

Compared to 500 mg of metformin, 20 mg and 40 mg of teneligliptin showed better antidiabetic effect, but 5 mg and 10 mg of teneligliptin showed poorer efficacy. Metformin (500–2,500 mg) is the first-line medication for treatment of T2DM (44). If the therapeutic effect of metformin is not ideal (HbA1c ≥ 7%), teneligliptin combined with metformin can achieve synergistic effect. As a note, the number of participants treated with metformin was limited (*n* = 35) in the included RCTs, which may lead to deviations for this study.

Bromocriptine was approved for the treatment of T2DM as an adjunct to diet and exercise to improve glycemic control by FDA in 2009 (45). It does not belong to popular antidiabetic drugs. Compared to four doses of teneligliptin, bromocriptine was poorer in most efficacy outcomes and better in reducing BMI, and showed a lower risk of hypoglycemia and a higher risk of GIAEs. As a note, limitation of the number of participants treated with bromocriptine (*n* = 25) may lead to deviation of the results.

We conducted a literature search and review about the meta-analyses containing teneligliptin, as summarized in **Table S3**. In previous studies (46, 47), efficacy and safety of teneligliptin were evaluated by very limited systematic reviews and traditional meta-analyses, which only included 10 (47) or 13 (46) RCTs. In the present study, 18 RCTs enrolling 3,290 patients with different interventions were included. There were also two network meta-analyses (48, 49) involving teneligliptin. However, teneligliptin was not the main evaluation object in the two network meta-analyses, and the outcome measure of evaluation of teneligliptin was very limited, which only included HbA1c (48) or incidence of GIAEs (49). Teneligliptin was the main evaluation object in the present Bayesian network meta-analysis, and eight outcome measures were included to systematically evaluate the efficacy and safety of teneligliptin.

In the present study, a serious search comprehensively covered the latest research findings, and independent study identification, selection, and data extraction were performed by two reviewers. Nevertheless, the heterogeneity and publication bias of included studies were not avoided. As a note, our inference was based on currently available data from a limited number of RCTs, and more large-scale, high-quality, and long-term clinical trials are needed to assess efficacy and safety of teneligliptin in the future.

## 5 Conclusion

In summary, efficacy and safety of teneligliptin in patients with T2DM were evaluated by Bayesian network meta-analysis of 18 RCTs in this study. Compared to sitagliptin, vildagliptin, metformin, bromocriptine, and placebo, teneligliptin displayed favorable efficacy and acceptable safety in the treatment of T2DM. Twenty milligrams or 40 mg per day could be chosen as the optimal dosage regimen for teneligliptin.

## Data availability statement

The original contributions presented in the study are included in the article/**Supplementary Material**. Further inquiries can be directed to the corresponding author.

## Author contributions

MZ: Investigation, Methodology, Writing – original draft, Data curation, Validation. RG: Data curation, Investigation, Methodology, Validation, Writing – original draft. GM: Conceptualization, Data curation, Formal analysis, Funding acquisition, Investigation, Project administration, Resources, Software, Supervision, Writing – review & editing, Validation.
