# Supplementary material for: Efficacy and safety of teneligliptin in patients with type 2 diabetes mellitus: a Bayesian network meta-analysis
Source: Front Endocrinol (Lausanne). 2023 Dec 18;14:1282584. doi: 10.3389/fendo.2023.1282584 (PMC10766708; doi:10.3389/fendo.2023.1282584)
Supplement: Supplementary file 2 [file Table_2.docx]

**Table S2** The sensitivity analysis of BW and BMI outcome.

| **Included study** | **I^2^ value** | |
| --- | --- | --- |
|  | **BW** | **BMI** |
| All Studies | 58.29% | 100 |
| Without Lee 2022 Study | 62.93% | NA |
| Without Ji 2021a Study | 61.56% | NA |
| Without Ji 2021b Study | 58.75% | NA |
| Without Nisha 2020 Study | NA | 100.00% |
| Without Kim 2019 Study | 58.91% | 100.00% |
| Without Agarwal 2018 Study | 54.32% | NA |
| Without Kadowaki 2018 Study | 54.35% | NA |
| Without Kadowaki 2017 Study | 42.62% | NA |
| Without Hong S 2016 Study | 60.18% | NA |
| Without Kadowaki 2014 Study | 59.79% | NA |
| Without Kadowaki 2013a Study | 59.92% | NA |
