# Supplementary material for: Efficacy and safety of teneligliptin in patients with type 2 diabetes mellitus: a Bayesian network meta-analysis
Source: Front Endocrinol (Lausanne). 2023 Dec 18;14:1282584. doi: 10.3389/fendo.2023.1282584 (PMC10766708; doi:10.3389/fendo.2023.1282584)
Supplement: Supplementary file 3 [file Table_3.docx]

**Table S3** The literature review about previous meta-analyses containing teneligliptin.

| **Literature** | **Study Type** | **Study Objectives** | **Comparisons items containing teneligliptin** |
| --- | --- | --- | --- |
| Li et al., 2018 | Traditional meta-analysis of 10 RCTs | To access the efficacy and safety of teneligliptin compared to placebo in patients with T2DM. | HbA1c, FPG, 2h PPG and AUC_0−2h_ for PPG, HOMA-β and HOMA-IR, incidences of overall AEs and hypoglycemia |
| Pelluri et al., 2023 | Traditional meta-analysis of 13 RCTs | To access the efficacy and safety of teneligliptin compared to placebo in patients with T2DM. | HbA1c, FPG, BW, incidence of hypoglycemia, cardiovascular outcomes |
| Ayers et al., 2017 | Network meta-analysis of 27 RCTs | To determine the efficacy and safety of liraglutide compared to DPP-4is as antidiabetics for Japanese patients with uncontrolled T2DM. | HbA1c |
| Wu et al., 2017 | Network meta-analysis of 165 RCTs | To systematically evaluate the effect of DPP-4is on GIAEs in patients with T2DM. | Incidence of GIAEs |

RCTs, randomized controlled trials; T2DM, type 2 diabetes mellitus; DPP-4is, dipeptidyl peptidase-4 inhibitors; FPG, fasting plasma glucose; 2h PPG, 2 h postprandial plasma glucose; AUC, area under curve from 0 to 2h; HOMA-β, Homeostasis model assessment-β; HOMA-IR, homeostatic model assessment for insulin resistance; AEs, adverse events; BW, body weight; GIAEs, gastrointestinal adverse events.
